# Medical Lectures

**Published:** 1844-05

**Authors:** 


MEDICAL LECTURES.

Several gentlemen are engaged in the course of Lectures at the Medical Institute announced for the Summer. The session is divided into two terms—the first extending to the 1st of July, the second beginning the 1st of September and closing with October. Doctors Buck, Hagan, and Ferguson deliver their lectures in the Spring division; Drs. Cobb, Gross and Miller will lecture during the Fall term, and Drs. Caldwell, Yandell, Bayless, and Colescott are engaged in both terms. Clinical instruction is delivered at the Hospital. Y.

